# Supraclinoid internal carotid artery blister-like aneurysms: hypothesized pathogenesis and microsurgical clipping outcomes

**DOI:** 10.1186/s41016-020-00226-w

**Published:** 2021-02-01

**Authors:** Shanwen Chen, Xin Chen, Bo Ning, Yong Cao, Shuo Wang

**Affiliations:** 1 Department of Neurosurgery, the Hospital of Shunyi District, No.3 Guangming Nan Street, Shunyi District, Beijing, 101300 China; 2 Department of Neurosurgery, Beijing Tiantan Hospital, Capital Medical University, China National Clinical Research Center for Neurological Diseases, No. 119 South 4th Ring West Road, Fengtai District, Beijing, 100050 China; 3 Department of Neurosurgery, Guangzhou Red Cross Hospital, Jinan University, No. 396 Tongfu Zhong Road, Haizhu District, Guangzhou, 510220 Guangdong China

**Keywords:** Blister-like aneurysm, Internal carotid artery, Microsurgical clipping, Morphology, Prognosis

## Abstract

**Background:**

Blister-like aneurysms (BLAs) on the supraclinoid segment of the internal carotid artery (ICA) are an enigma of cerebrovascular disease. Neither has a definite pathogenesis been so far identified, nor have uniform treatment guidelines been established for them. Our aim was to develop a hypothesis regarding the evolution of BLAs according to their macroscopic morphologies and to evaluate the efficacy of microsurgical clipping.

**Methods:**

The clinical data and morphological features of 15 consecutive patients with 16 BLAs on the supraclinoid ICA were retrospectively reviewed. The treatment strategies were analyzed, and functional outcomes were evaluated using the modified Rankin scale (mRS). Favorable outcomes were defined as a mRS score of 0–2.

**Results:**

Morphologically, aneurysm growth with expansion of the aneurysm neck before the surgical procedure occurred in two ruptured and one unruptured aneurysm. Daughter bleb formation was observed in two ruptured and five unruptured aneurysms. A varied degree of parent artery sclerosis was observed in nine patients. Thirteen patients were treated with direct surgical clipping, one patient was treated with clipping and wrapping, and the remaining patient was treated with an encircling clipping graft. Favorable and unfavorable outcomes were observed in 13 and two cases, respectively. Follow-up angiograms revealed 4 cases of stenosis with respective degree of mild, 30%, 50%, and 80% without any neurological dysfunction.

**Conclusions:**

We suggest a hypothesis that BLAs on the supraclinoid ICA may share different evolving mechanisms between ruptured and unruptured lesions. A majority of them can be reliably and safely obliterated by direct clipping technique, except for the aneurysms accompanied with severely atherosclerotic parent walls.

## Background

In 1969, Sundt and Murphey first described a sessile lesion at a nonbranching site on the dorsal wall of the internal carotid artery (ICA) and labeled it as a “blister” aneurysm [1]. Many Japanese researchers further reported numerous patients harboring ICA blister-like aneurysms (BLAs) during the 1980s–2000s. Before 2000, a lack of sufficient cognition existed about the nature of this type of aneurysm; thus, a variety of unfavorable complications were perioperatively associated with surgical procedures, including intraoperative rebleeding, postoperative recurrence, and ischemia. These frustrating cases precisely exemplified the false nature of the ruptured BLAs, especially in four published histopathological reports [2–5]. Nonetheless, we know nothing about how BLAs evolve at present. To date, different treatment modalities have been proposed, but none has established superiority to the others. Without internationally accredited guidelines, neurosurgery centers worldwide performed the procedures in which they were most skilled to treat BLAs. In the present study, we retrospectively reviewed 15 cases of 16 BLAs on the supraclinoid ICA, consisting of six ruptured and ten unruptured aneurysms, to examine their heterogeneous morphologies, develop a hypothesis regarding their evolution, and evaluate the therapeutic effects of microsurgical clipping.

## Methods

This clinical observational study was performed between January 2012 and June 2017. We screened an electronic database for all ICA aneurysms treated with microsurgical techniques to identify BLAs. BLAs were defined as shallow, broad-based aneurysms arising from nonbranching sites of the supraclinoid ICA on diagnostic cerebral angiograms and further confirmed by surgical inspection.

The patients’ clinical examinations and imaging studies, operative and angiographic reports, intraoperative photographs or videos (if available), and in- and outpatient records were reviewed. We documented the clinical characteristics, including sex, age, history, symptoms at presentation, treatment modalities, complications, and outcomes; diagnostic angiographic features, including aneurysm size, side, location, and contour appearance; and intraoperative findings, such as wall heterogeneity and ICA sclerosis, for each patient. Initial digital subtraction angiography (DSA) was performed to evaluate the aneurysm and surrounding vessels. Postoperative DSA or computed tomographic angiography (CTA) was performed to document aneurysm obliteration and the degree of parent vessel stenosis. Postoperative CT scans were performed to assess subsequent ischemic occurrence. Follow-up imaging was performed at least 3 months after surgery by DSA or CTA. Clinical outcomes were measured at least 1 year later with the modified Rankin scale (mRS). Patients achieving an mRS score of 0–2 were considered to have favorable outcomes.

All enrolled patients were initially treated with microsurgery. Generally, a pterional or lateral-frontal craniotomy was performed for ruptured and unruptured BLAs, respectively. Regarding the former procedure, the Sylvian fissure was opened to expose the lateral side of the ICA from outward to inward, and thereby, proximal control of the parent artery was obtained. For the latter procedure, the frontal lobe was gently elevated along the anterolateral cranial base to approach the optic nerve and chiasm. The anterior clinoid process was carefully drilled and removed when necessary. An epidural approach was sometimes performed to prevent premature disturbance to the aneurysm itself. The carotid and optic cisterns were exposed, and the arachnoid was sharply dissected to fully expose the supraclinoid segment of the ICA, the anterior cerebral artery A1 segment and the middle cerebral artery M1 segment. Appropriate aneurysm clips were selected for the BLAs based on their site, size, and shape. The clip was placed parallel to the carotid, purposely gripping a part of the normal carotid wall beyond the aneurysm and producing mild stenosis of the ICA. A temporary clip played a role in decreasing intra-aneurysm tension to facilitate clipping. Electrophysiological monitoring, including motor evoked potentials and somatosensory evoked potentials, was recorded intraoperatively to assess the cerebral perfusion. To verify the stability of the clip and the absence of errhysis in the operating field, the anesthesiologist was asked to briefly suffocate the patient by ceasing mechanical ventilation for 45 s.

## Results

### Clinical characteristics of patients

We identified 16 aneurysms in 15 patients with a diagnosis of supraclinoid ICA BLA. A female predominance was observed, with ten women versus five men and a mean age of 50.3 years (range 31–73 years). Six patients had a history of hypertension. Six patients (40%) presented with aneurysmal subarachnoid hemorrhage (aSAH), of whom four were in the acute phase and two were in the chronic phase. At admission, the Hunt-Hess grade ranged from 1 to 2, and the Fisher grade ranged from 1 to 3. Nine patients (60%) were incidentally identified as having aneurysms due to nonspecific pathologies or symptoms.

### Macroscopic morphologies of the aneurysms

**Fig. 1:**
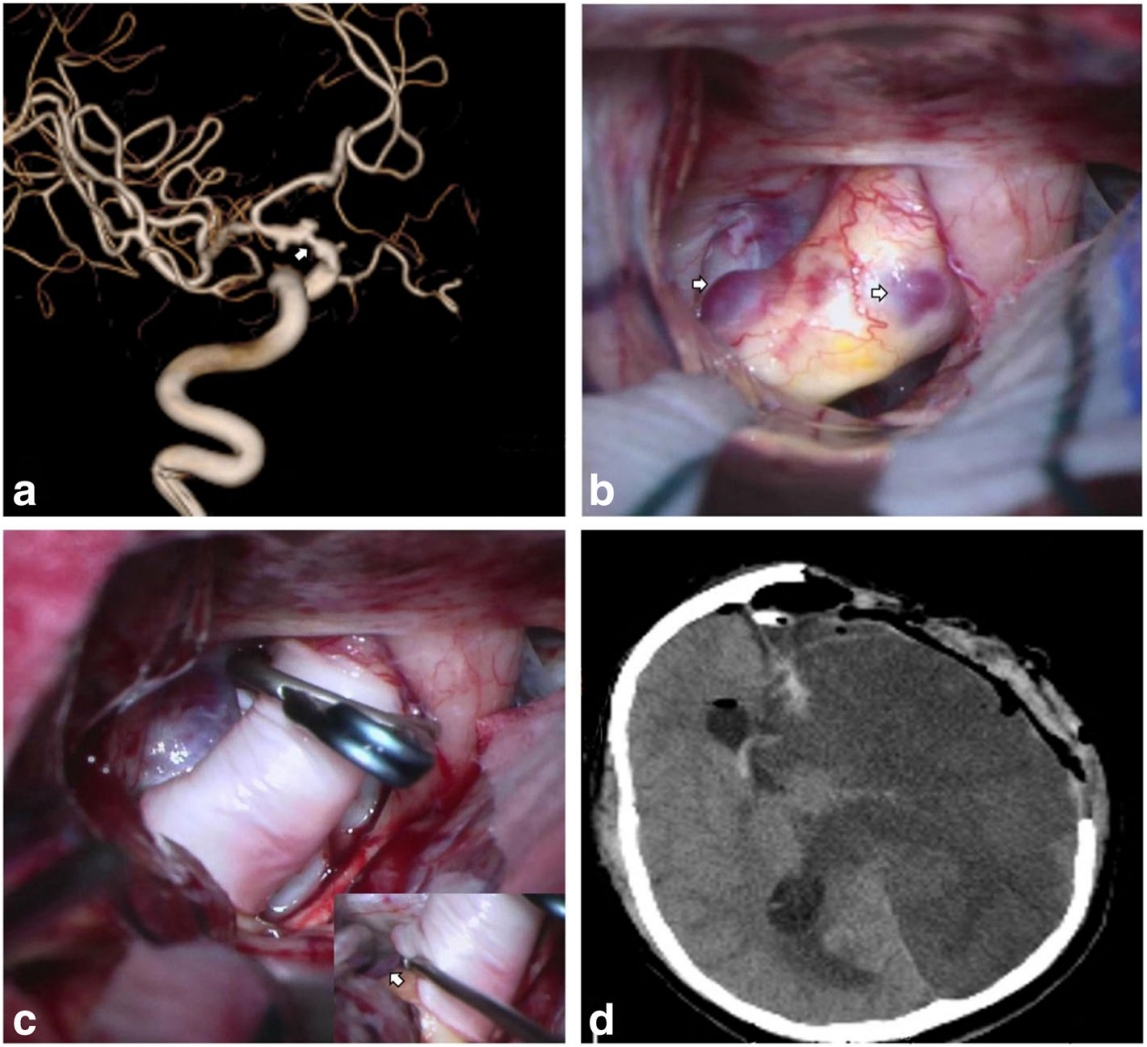
Case 8. **a** Preoperative 3D DSA demonstrated two neighboring ICA BLAs and notably stenotic lumen (white arrow). **b** Intraoperative findings showed the macroscopic features of both BLAs (white arrow) and entirely sclerotic parent wall. **c** Both BLAs were obliterated and the inset showed patency of the left anterior choroidal artery (white arrow). **d** Postoperative CT revealed left hemispheric infarct

The morphological features of the aneurysms are shown in Table 1. All BLAs were located on the nonbranching sites of the ICA anterior, anteromedial, anterolateral, or lateral wall. Of all the BLAs, six were on the right side, and ten were on the left side. Nine involved the C6 segment, four involved the C6-7 junction, and three involved the C7 segment adjacent to the origin of the anterior choroidal artery. One patient had two neighboring BLAs, just distal to an obviously stenotic lumen on diagnostic DSA images (Fig. 1, case 8).

**Table 1:** Clinical and morphological characteristics of the patients

Patient	Age (years)/sex	Symptoms	Location	Size (neck × dome) mm	Growth	Angiography	Intraoperative visualizations	ICA sclerosis
1	51/M	SAH	C6 anterior	3.64 × 2.16	No	Hemispherical	Bright red, covered with clot	Medial wall
2	55/F	SAH	C7 anterolateral	4.71 × 3.93	No	Hemispherical	Reddish	NM
3	52/M	SAH	C6-C7 anteromedial	4.65 × 3.45	No	Irregular, bleb at dome	Bright red, covered with clot	Lateral wall
4	56/M	SAH	C6-C7 anterior	4.58 × 4.45	No	Irregular, bleb at the anteromedial wall	Reddish, covered with clot	Local wall
5	43/F	SAH	C6-C7 anterior	3.27 × 2.88	Yes	Hemispherical	Reddish, covered with clot and fibrinous tissue	No
				8.24 × 6.63		Saccular		
6	58/F	SAH	C6 anterior	Sessile	Yes	Tapered	Reddish, whitish rupture gap adhered to the left optic nerve	Lateral wall
				2.58 × 1.54		Tapered		
7	73/M	Headache, dizziness	C6 anterolateral	3.98 × 3.42	No	Irregular, bleb at the anteromedial wall	Reddish, bright red bleb	NM
8	59/M	Mild brain infarct	C6-C7 anteromedial	4.68 × 2.81	No	Both hemispherical, proximal ICA stenosis	Bright red and reddish heterogeneity	Entire supraclinoid wall
			C7 lateral	2.76 × 1.79	No		Reddish base, bright red dome	
9	39/F	Recurrent dizziness	C6 anterolateral	2.47 × 1.94	No	Irregular, bleb at the anterolateral wall	Reddish, bright red bleb	Medial and lateral wall
10	47/F	Headache	C6 anterior	2.69 × 1.70	No	Hemispherical	Bright red	Distal anterior wall
11	32/F	Headache	C6 anterolateral	4.66 × 2.70	No	Irregular, bleb at the medial wall	Reddish, bright red bleb	No
12	31/F	Headache	C6 anterior	4.69 × 3.89	No	Hemispherical	Reddish base, bright red dome	No
13	32/F	Headache, diplopia	C7 lateral	4.13 × 3.26	No	Irregular, bleb at the posterolateral wall	Bright red, an extremely thin bleb	Proximal anterior wall
14	64/F	Dizziness	C6 anterolateral	2.46 × 2.07	Yes	Hemispherical	Reddish base and bleb	Lateral and distal anterior wall
				6.46 × 4.31		Irregular, bleb at the anteromedial wall		
15	63/F	Headache, dizziness	C6 anterior	2.75 × 1.55	No	Hemispherical	Reddish	NM

The Bouthillier classification of internal carotid artery segments: C6, ophthalmic; C7, communicating

*F* Female, *M* Male, *NM* Not mentioned

**Fig. 2:**
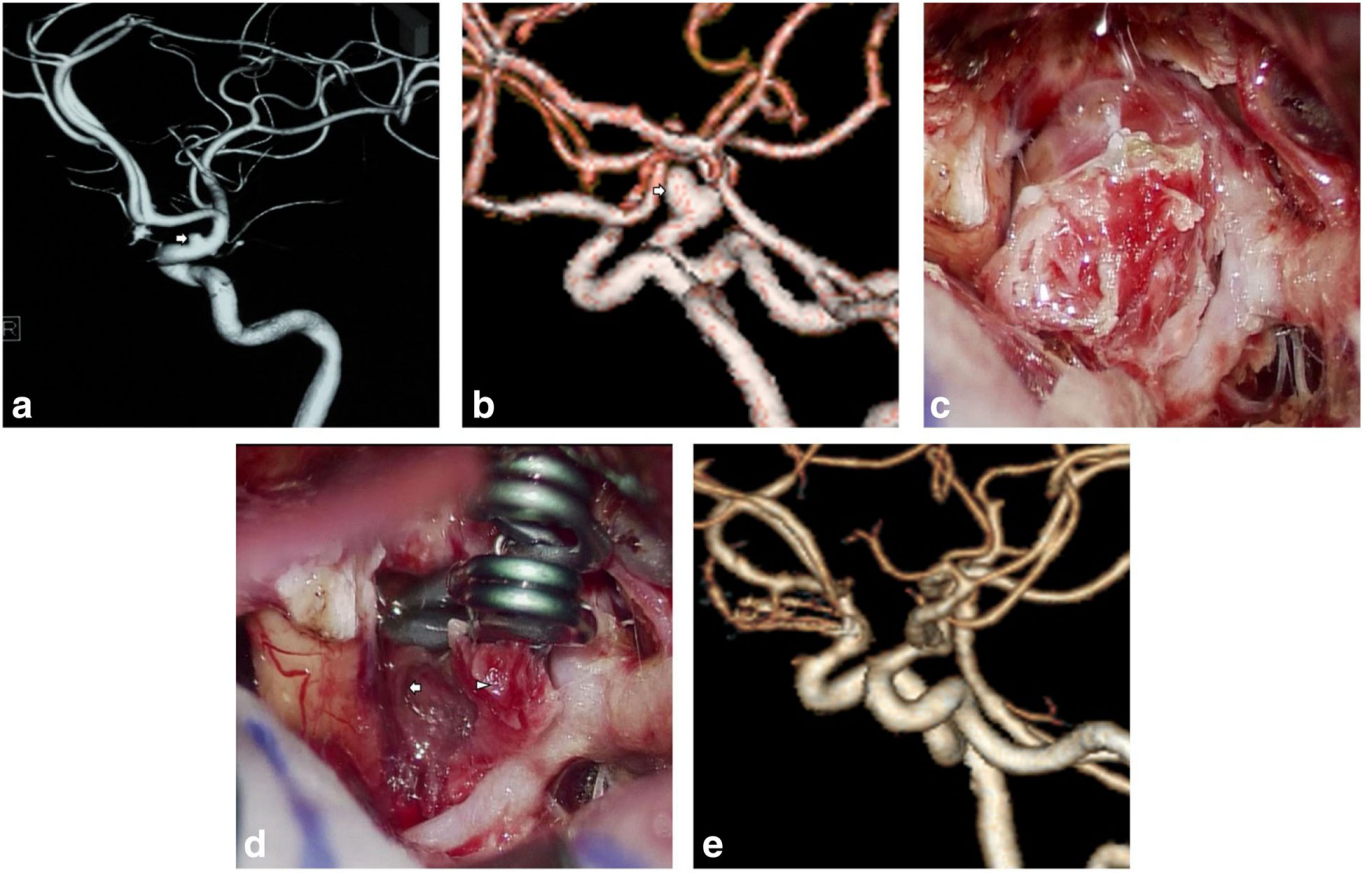
Case 5. **a**, **b** Preoperative angiography demonstrated a tapered BLA and its rapid growth into saccular configuration (white arrow). **c** This anteriorly projecting aneurysm was tightly adhered to the frontal lobe. **d** The aneurysm was composed of a thickened false membrane (arrowhead) and a much thinner true wall (arrow). **e** Postoperative CTA showed complete obliteration of the aneurysm

**Fig. 3:**
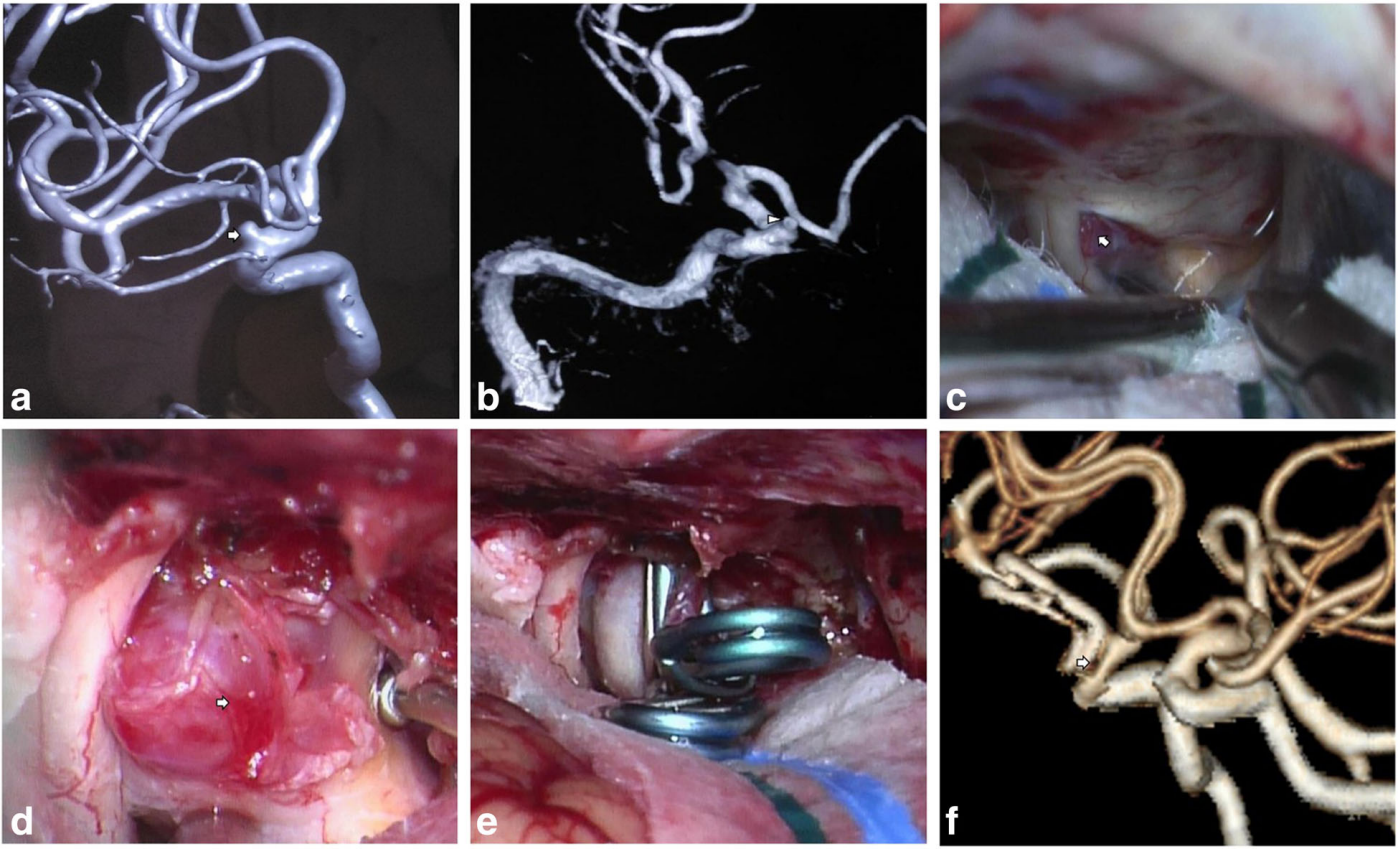
Case 14. **a**, **b** Preoperative 3D DSA demonstrated a hemispheric BLA (white arrow) and its growth accompanied with a daughter bleb (arrowhead). **c** This medially projecting BLA was barely visible underneath the optic nerve and the anterior clinoid process (white arrow). **d** The aneurysm and its translucent bleb (white arrow) originated from a partially sclerotic parent wall. **e** The aneurysm was obliterated. **f** Postoperative CTA showed 50% stenosis (white arrow) of the parent artery

Aneurysm growth with neck dilation observed on diagnostic angiography occurred in two ruptured and one unruptured BLA. One BLA exhibited rapid growth into a saccular configuration and notable neck expansion within a short interval of 11 days after the ictus of aSAH. Under a microscopic view, we detected that the aneurysm sac was composed of a thickened false membrane covering a larger area on the distal side and a much thinner true wall occupying a smaller area on the proximal side, and the membrane and wall were connected with each other (Fig. 2, case 5). The other BLA ceased growing in the latter three follow-up DSAs and the aneurysm base expanded only slightly (case 6). One unruptured BLA developed accompanied by a daughter bleb forming within half a year (Fig. 3, case 14). Daughter bleb formation was observed in two ruptured and five unruptured BLAs, corresponding to the thinner aspect of the aneurysm wall, through which vortices of blood flow were clearly seen in operation. Acute ruptured BLAs were packed with clotted and fibrotic tissue on the dome, as depicted in previously published literature, whereas two chronic aneurysms had a smooth and reddish (not bright red) wall. Most unruptured aneurysms had heterogeneous wall thicknesses and were focally bright red, and the rest were reddish, even if no daughter bleb existed.

A varied range of sclerosis in the supraclinoid ICA existed in nine patients, even in younger patients without a history of hypertension and hyperlipemia; no atheromatosis was visualized in three cases, and the remaining three cases had no related depictions. Of nine patients with atheromatosis, eight had a focal sclerotic plaque immediately adjacent to the origin of the BLA, generally on the medial or lateral side but sometimes on both sides (Fig. 3). One patient had a completely atherosclerotic supraclinoid portion of the ICA, on which double BLAs were situated (Fig. 1, case 8).

### Microsurgical treatment and patient outcomes

All patients were microsurgically treated, and their treatment modalities and outcomes are shown in Table 2. Of the 13 aneurysms successfully treated with initial clipping reconstruction, four were reconstructed using straight clips (single or stacked) with the blades oriented perpendicular to the axis of the parent ICA and five were clipped using curved clips with the tips parallel to the parent ICA. Multiple stacked side-angled clips were applied in two cases (Fig. 2, case 5), and right-angled clips were required for the remaining two cases to obtain aneurysm obliteration (Fig. 3, case 14).

**Table 2:** Microsurgical treatment and outcomes of the patients

Patient	Treatment	IOR	ICA stenosis	Ischemia	Vasospasm	Discharge mRS	F/U mRS	F/U (m)
1	3 straight clips (stacked), wrapping	Yes	80%	No	Yes	1	0	49
2	3 side-angled clips (stacked)	No	No	No	No	0	0	54
3	1 straight clip	No	No	No	No	1	0	18
4	2 straight clips	No	NA	Yes	Yes	4	3	20
5	2 side-angled clips (stacked)	No	No	No	No	0	0	17
6	1 straight clip	No	No	No	No	0	0	16
7	2 curved clips	No	No	No	No	0	0	47
8	Circumferential wrapping, 1 fenestrated clip	No	NA	Left global infarct	NA	5	5	13
		No						
9	1 curved clip	No	30%	No	No	0	0	31
10	1 curved clip	No	No	No	No	0	0	21
11	1 curved clip	No	No	No	No	0	0	20
12	1 curved clip	No	Mild	No	No	0	0	24
13	1 straight clip	No	No	No	No	0	0	17
14	2 right-angled clips (stacked)	No	50%	No	No	0	0	18
15	1 right-angled clip	No	No	No	No	0	0	44

*IOR* Intraoperative rupture, *NA* Not applicable, *F/U* Follow-up

Case 1 was the only instance of intraoperative rebleeding in this series, attributed to the part of neck avulsion at the moment that the clip blades were closed, though the proximal ICA had been temporally controlled. Thereafter, the other two stacked straight clips were applied to catch the normal ICA wall and reconstruct the parent vessel. The profuse bleeding was successfully resolved. A slice of temporal muscle was applied to strengthen the avulsed neck. Severe ICA stenosis expectedly emerged. However, electrophysiological monitoring surprisingly evidenced no decline compared to the data before the clipping procedure, probably owing to contralateral ICA or vertebrobasilar blood compensation.

In case 8, double BLAs neighbored each other at the C6-C7 junction anteromedial wall and C7 lateral wall, the former having a broader base than the latter. The concomitant proximal ICA stenosis was distinctly demonstrated on DSA images. Under the microscopic vision, the entire supraclinoid ICA manifested xanthochromia, indicating an atherosclerotic and tough wall. We tried to apply a curved clip to address the larger aneurysm. However, the clip slipped off while grasping a small atherosclerotic wall. We replaced the clip with a larger-sized one to grasp more of the parent artery wall and to prevent slippage once again, and iatrogenic stenosis obviously arose. Given the probability that secondary cerebral ischemia would result from the iatrogenic stenosis if direct parallel clipping was performed to both BLAs, as well as from the pre-existing proximal ICA stenosis, we ceased direct clipping and decided to circumferentially wrap both BLAs with a graft of an artificial membrane of 1 cm width and then to reinforce the wrapping material with a cross-vascular clip gripping it tightly. After temporary occlusion of the proximal ICA to reduce aneurysm tension, the protocol was scrupulously performed, sacrificing the underdeveloped ipsilateral posterior communicating artery while maintaining the patency of the ipsilateral anterior choroidal artery (Fig. 1). Electrophysiological monitoring remained normal. However, the patient showed poor consciousness and right muscle power weakness after being transferred to the neurosurgical intensive care unit. An urgent CT scan revealed a hypodensity in the ipsilateral corona radiata and the centrum semiovale. In view of ischemic occurrence, 3H therapy was exerted to increase cerebral perfusion. The patient deteriorated with complete right-side paralysis, and a second CT scan performed the next morning showed left hemispheric infarction (Fig. 1). Thereafter, a second external decompression and a third internal decompression had to be carried out to save his life.

Overall, 13 patients (87%) in this series had a favorable clinical outcome with an mRS score of 0–1. Their follow-up images showed thorough obliteration of aneurysms. Two patients (13%) were disabled with new permanent neurological deficits. Good outcomes were observed in five of six patients presenting with aSAH (83%). Postoperative vasospasm developed in two of four patients with acute aSAH. One patient (case 4) experienced a right middle cerebral artery stroke and resultant left-side extremity hemiparesis, attributed to severe vasospasm. His prognostic mRS score was 4 when discharged, and this value improved to 3 at the last follow-up. The other patient (case 1) experienced mild vasospasm and, as a result, short-time somnolence. His postoperative CTA revealed complete obliteration of the lesion, accompanied by left ICA stenosis up to 80%. Follow-up CTA at 6 months revealed the same results. Nonetheless, the patient avoided ischemic attacks due to a good compensatory blood flow by postoperative opening of the anterior communicating artery. He had a good recovery with discharge and follow-up mRS scores of 1 and 0, respectively. Of nine patients with unruptured BLAs, eight maintained an intact neurological status. In contrast, case 8 exhibited an mRS score of 5 at discharge because of left massive hemispheric infarction, and the last mRS measurement showed no improvement at the 1-year follow-up. Moreover, three patients had iatrogenic ICA stenosis, varying from mild stenosis in case 12 to 30% stenosis in case 9 and 50% stenosis in case 14.

## Discussion

### Hypothesized pathogenesis based on the morphological analysis

In general, ruptured BLAs exhibit unique features of their own, namely, a thin, translucent, and friable wall, and the concomitant pathological parent artery. BLAs are considered to be formed from disruption of the intima and media and subsequent laceration of the adventitia, which is covered with blood clots and fibrin, according to well-known histopathological findings on autopsy [2] and from live specimens recently obtained from two patients [4]. Therefore, BLAs are viewed as false aneurysms in nature, a definition that has been widely approved beyond all doubt. In this situation, the parent arterial wall, including three layers of organized structures, is penetrated from the inside out and may be destroyed in an instant by impingement of the blood flow. If one lesion is not surgically or interventionally disposed of in time, it may have a chance to grow within a short period or will enter the chronic stage with reorganization of the aneurysm wall. The question is, however, whether ruptured BLAs share the same mechanism leading to rupture. Atheromatosis, dissection, and hemodynamic stress have been regarded as three major etiologies, but it is still unknown how they interact to yield BLAs and how BLAs evolve into large aneurysms.

Atherosclerosis was deduced to initiate BLAs due to laceration of the ICA wall, caused by ulceration and penetration into the internal elastic lamina (IEL) [2, 6]. In the present study, complete sclerosis occupied the whole terminal ICA distal to the ophthalmic artery in case 8, where both BLAs protruded out of the sclerotic wall. In contrast, eight other cases had eccentric sclerosis on the ICA walls, which were medially or laterally adjacent to the aneurysms, and three young patients had no signs of atherosclerosis. We searched ten cases in the previous literatures harboring BLAs on the supraclinoid ICA that originated thoroughly from the sclerotic plaque, according to the illustrated figures or the authors’ literal narrations [3, 5, 7–13]. Therefore, we suggest, based on the ubiety between both lesions, that BLAs should be classified into 3 groups. Group 1 contains lesions situated fully or mostly at the atherosclerotic site, whose initiation may primarily be associated with atherosclerotic ulceration and penetration. Group 2 contains lesions originating from the junction of the sclerotic plaque and a normal portion of the vessel, where the IEL is abruptly terminated and the media disappears as well [2]. As a result, the arterial wall may be vulnerable under hemodynamic stress. Group 3 contains lesions with no sclerotic occurrence in addition to the aneurysm, which is not rare in younger individuals and should be completely attributed to the influence of hemodynamic stress. In short, different groups might exhibit different pathogeneses.

We noted that four aneurysms in the ruptured group had a regular hemispheric or tapered contour, whereas the other two showed an irregular appearance with daughter blebs. Coincidentally, five of ten aneurysms in the unruptured group gave rise to daughter blebs as well. Daughter blebs are commonly regarded as existing breakdown points in an aSAH or latent points in unruptured aneurysms, and the evolution of daughter blebs is a time-consuming process due to hemodynamic stress. Therefore, we surmise that different mechanisms likely lead to rupture in BLAs. Some BLAs are believed to undergo instantaneous tearing of whole layers of the parent wall, thus facilitating false aneurysms. This so-called primary rupture consists of initial evolution of rupture and subsequent growth, which is different from that of common saccular aneurysms. We believe that this theory can reasonably explain why the initial angiogram was negative and repeated procedures were needed to verify the source of SAH in many previously reported cases. It should be noted that rapid growth of a BLA into saccular configuration with neck expansion is likely associated with a major area of focal subadventitial dissection, which is derived from instantaneous laceration of the parent wall as soon as the aneurysm is initiated. Dissection has been shown in two recent case reports with definite proof by histological examination and high-resolution magnetic resonance imaging [4, 9]. Additionally, some BLAs undergo laceration of two layers, the intima and media, leaving the adventitia intact. In this circumstance, the aneurysm might grow slowly, or even yield a new bleb, and ultimately collapse until it cannot endure hemodynamic stress. We refer to this process as “secondary rupture.”

### Microsurgical clipping

Microsurgical clipping still merits consideration as the principal modality for treating ICA BLAs [14], as endovascular treatment with or without stents and flow diversion devices (FDDs) have not yet shown substantial superiority. Three recent systematic reviews with meta-analyses compared the advantages and disadvantages of microsurgery and endovascular therapy [15–17]. In general, microsurgery offers a superior obliteration rate at the cost of a higher complication rate due to a higher rupture rate. In contrast, endovascular therapy offers superior safety at the cost of a lower obliteration rate and higher retreatment rate due to incomplete dense packing with a residual neck. However, both therapies did not statistically differ in clinical outcomes [15, 16]. Specifically, stent-assisted coiling had high recurrence rates of 25%, 40%, and 50% in three independent studies [18–20]. Considering its inefficiency, Szmuda et al. stated that stent-assisted coiling appeared to be the least beneficial method [16]. FDDs represent a promising option to treat BLAs. Rouchaud et al. compared the efficacy of several reconstructive endovascular modalities, including FDDs, stent placement, and stent-assisted coiling, and concluded that FDDs had a higher rate of mid- to long-term complete occlusion than other reconstructive techniques (90.8% versus 67.9%) and a lower rate of retreatment (6.6% versus 30.7%) [21]. However, FDDs also have the disadvantages of a low initial obliteration rate and early rebleeding. Adjunctive use of coiling can achieve a higher incidence of immediate occlusion of BLAs [22]. Moreover, dual anti-platelet therapy is another challenging factor for the use of FDDs in the acute phase of aSAH.

Microsurgical clipping consists mainly of direct parallel clipping, clipping on the wrap, and placing an encircling clip graft. In our experience, anterior clinoidectomy plays an important role in treating a premature aneurysm rupture. This technique can provide adequate space to gain proximal control of the supraclinoid ICA if the aneurysm extends very close to the distal ring of the ICA. Compared to the subdural approach, epidural clinoidectomy seems to be much safer because of no retraction of the frontal lobe and less disturbance to the arachnoid before reaching the aneurysm. When clearing the clot in the carotid and optic cisterns, sharp dissection is absolutely advocated, and the thrombus capping the rupture dome must be cautiously avoided. Temporary proximal occlusion is inevitably needed to decrease the tension of the friable wall to avoid neck avulsion. The application of an aneurysm clip is a pivotal step, and picking a clip with an appropriate shape and size should be seriously considered. An angled, slightly curved, or side-angled clip, rather than a straight clip, capable of catching a portion of the healthy arterial wall with the blades parallel to the axis of the ICA trunk, is usually selected. If the initial clip does not stay in situ and slips toward the aneurysm dome, a second understacked clip should be placed to secure the aneurysm base.

In the present study, six cases with ruptured BLAs achieved direct parallel clipping successfully, although one of them underwent an accidental rupture intraoperatively. In this incipient case, a straight clip was initially picked out and applied parallel to the artery’s axis. Due to the incorporation of less healthy tissue, the clip unexpectedly slipped off as soon as its two blades closed, and the aneurysm base tore. Another two stacked straight clips were urgently placed beneath the initial one. Although the patient had a good clinical outcome, it seemed much better that intraoperative avulsion would have been avoided if a curved clip was initially selected. Regarding the unruptured BLAs shown in our nine cases, their heterogeneous walls were virtually not as fragile as those of the ruptured BLAs, were able to tolerate clipping torsion, and were generally not easy to rupture. There are no reports related to intraoperative rupture of unruptured BLAs in previous studies [23–25].

In our series, clipping on the wrap was not used because successful direct parallel clipping was conducted in 14 of 15 cases, and the remaining one case was not suitable due to the configuration of both BLAs. Encircling the clipping graft was carried out after failing to occlude the aneurysm by direct clipping. We prefer encircling the clipping graft if a wide atherosclerotic wall exists beside the aneurysm, which may lead to incomplete clipping or clip slippage when gripping a small part of the wall beyond the aneurysm, or if the patient has an extremely broad-base aneurysm or multiple aneurysms, which may result in excessive ICA stenosis and possibly secondary cerebral ischemia when applying direct parallel clipping. As demonstrated by case 8 in the present study, two neighboring BLAs were wrapped by a sheet of artificial membrane and held by a fenestrated clip. Thereafter, they were perfectly obliterated. However, a delayed hemispheric infarct took place in this case, probably due to loss of patency of the parent vessel, deriving from a too-tight constraint on the arterial lumen plus pre-existence of proximal stenosis, or plaque disruption when the initial grabbing of side wall to maintain clip position or the later temporary occlusion of the proximal ICA was performed. For this patient, parent vessel trapping and bypass would have been the best strategy.

### Shortcomings of microsurgical clipping

As diagnostic angiography reveals only the vessel lumen rather than its wall, it is difficult to preoperatively ascertain the extent of the involved arterial wall that might be included in the microsurgical reconstruction. When it is difficult to predict intraoperatively the exact territory of the diseased artery, some surgery-related complications arise. Intraoperative rebleeding was ever the most lethal and disastrous accident, which was commonly due to partial or full neck avulsion when closing the clip blades [3, 5, 26, 27], clip slippage [27], and manipulation of clots that were typically on the dome of a BLA. Its inherent nature of fragility must be the determinant factor causing procedure-related rupture. Furthermore, focal tissue edema provoked by hemorrhagic injury at the transitional zone aggravates its fragility as well. Once the accident occurs, the surgeons, anesthesiologists, and instrument nurses will be fully engrossed in it. Some effective measures, for instance, suturing the tear with reinforcement by encircling wrapping and clipping [5, 8, 11, 25, 26], stacked clips as those used in our case 1 [6, 27], and vascular closure staple clips [28], will most likely guarantee a successful operation. Therefore, the rate of intraoperative rupture was reported to be remarkably declined [23, 29–31]. However, in cases with severely atherosclerotic parent walls, the abrupt histological transition from the sclerotic ICA wall to the fragile aneurysm neck is more likely to result in laceration of the wall because of clip torsion [32]. Among ten cases with BLAs protruding from the sclerotic plaque, five suffered from complete neck avulsion [3, 5, 8, 11], one was revised to an encircling silicone sheet clip before rebleeding [12], and the remaining four achieved success [7, 9, 10, 13]. In that situation, direct parallel clipping is extremely dangerous; thus, other rescue remedies, such as bypass and wrap-clipping, should be considered.

Although parallel clipping may result in a certain degree of stenosis of the ICA in the angiogram, angiographic stenosis does generally not trigger a hemodynamic decrease [26, 30, 31, 33]. Even if serious stenosis actually exists, the contralateral or vertebrobasilar blood supply, if good compensation presents, is capable of providing adequate perfusion. In our series, case 1 encountered such a situation in which the neurological function remained intact due to good blood compensation, although postoperative and follow-up angiograms showed severe stenosis of ICA up to 80%. If a certain stenosis of the parent vessel cannot be avoided, we will perform electrophysiological monitoring to evaluate variations in cerebral function following fluctuating blood flow.

The causes of postoperative recurrence are thought to be slippage of the clip, incomplete clipping, or de novo growth of the aneurysm due to insufficient inclusion of the adjacent wall of the parent artery within the clip blades [27]. Retreatment is definitely needed in cases of recurrent bleeding. To administer a second therapy, individual strategies, such as reclipping, surgical or endovascular ICA trapping, high-flow bypass, stent placement only, or stent-assisted coiling, are rendered [6, 7, 23, 34, 35]. In our series, angiographic recurrence or postoperative clinical rebleeding did not occur during the follow-up period.

### Limitation of the study

First, this investigation was a retrospective and observational study including a small number of cases in a single center. A smaller sample may lack persuasion to strongly support our hypothesis regarding BLA evolution. More cases, including BLAs located at atypical sites, will be enrolled to confirm the hypothesis in the next step. Second, a histopathological examination to support the hypothesized evolution mechanisms was not performed due to other uses of the surgical specimen from case 5. Third, more than half of the aneurysms belonged to unruptured groups; thus, the degree of operative difficulty might have been correspondingly mitigated.

## Conclusions

In summary, ruptured and unruptured BLAs on the supraclinoid ICA exhibit different morphological features that might suggest different evolving mechanisms. Although different therapies can be implemented, each neurosurgery center should perform the procedures in which they are most skilled to benefit patients. We suggest that direct clipping is still a reliable modality to treat BLAs with mild or no atherosclerosis of the parent vessel and often yields a good outcome. However, in cases with severely atherosclerotic parent walls, this modality is very difficult and generally dangerous, and an assessment of the aneurysm itself and the parent artery should be comprehensively performed to help determine an appropriate alternative solution.

## Data Availability

Data sharing is not applicable to this article as no datasets were generated or analyzed during the current study.

## Abbreviations

BLAs: Blister-like aneurysms
SAH: Subarachnoid hemorrhage
ICA: Internal carotid artery
mRS: Modified Frankin scale
DSA: Digital subtraction angiography
CTA: Computed tomographic angiography
IEL: Internal elastic lamina
FDDs: Flow diversion devices
